# Detoxication mechanisms of Radix *Tripterygium wilfordii* via compatibility with Herba *Lysimachia christinae* in S180-bearing mice by involving Nrf2

**DOI:** 10.1042/BSR20180429

**Published:** 2018-07-13

**Authors:** Jun-Ming Wang, Hong Cai, Jin-Hua Li, Rong-Xing Chen, Yue-Yue Zhang, Jin-Yang Li, Ning-Ning Ding, Chen Liu, Ying Cui

**Affiliations:** 1 College of Pharmacy, Henan University of Chinese Medicine, Zhengzhou 450046, China; 2 Collaborative Innovation Center for Respiratory Disease Diagnosis and Treatment and Chinese Medicine Development of Henan Province, Henan University of Chinese Medicine, Zhengzhou 450046, China

**Keywords:** Antioxidant genes, Compatibility detoxication, Hepatotoxicity, Lysimachia christinae Hance, Nrf2, Tripterygium wilfordii Hook F

## Abstract

The combined administration between Radix *Tripterygium wilfordii* Hook F (LGT) and Herba *Lysimachia christinae* Hance (JQC) belongs to mutual detoxication compatibility of seven emotions in traditional Chinese medicine (TCM) theory. However, until now, the compatibility detoxication mechanisms remain unknown. The present study was undertaken to observe detoxication mechanisms of LGT through compatibility with JQC in tumor-bearing mice by involving NF-E2-related factor 2 (Nrf2)-mediated antioxidant defenses. In addition, influence of compatibility on antitumor activity was also investigated here. Our results demonstrated that compatibility with JQC administration significantly reversed LGT-elevated serum alanine/aspartate transaminase (ALT/AST) levels and alleviated hepatocytes’ swelling or degeneration damage, and at the ratio 2/1 (LGT/JQC) produced the strongest detoxication effect. Besides, compatibility with JQC administration reversed not only LGT-elevated hepatic malondialdehyde (MDA) and tumor necrosis factor-α (TNF-α) but also the LGT lowered GSH, glutathione-s transferase (GST), glutathione peroxidase (GPx), superoxide dismutase (SOD), catalase (CAT), and interleukin (IL)-10 levels. Furthermore, compatibility with JQC administration significantly up-regulated protein expression of Nrf2 and mRNA expression of it regulated downstream antioxidant genes such as heme oxygenase-1 (*HO-1*), NAD(P)H: quinone oxidoreductase-1 (*NQO1*), and glutamate cysteine ligase catalytic subunit (*GCLC*). In addition, compatibility with JQC further decreased LGT-decreased tumor weight and at the ratio 2/1 (LGT/JQC) also exerted the strongest synergistic effect. Collectively, through compatibility with JQC exerted detoxication effect on LGT-induced hepatotoxicity and the mechanisms could be at least partly attributed to up-regulation of Nrf2 and its downstream signals, thereby enhancing antioxidant defenses, and inhibiting lipid peroxidation, oxidative stress, and inflammation. Additionally, at the ratio 2/1 (LGT/JQC) exerted the strongest effects on both detoxication and synergism.

## Introduction

Compatibility is one of the characteristics and advantages of traditional Chinese medicine (TCM) [1–3]. TCM compatibility mainly contains the seven emotions including using a single drug, mutual reinforcement, mutual assistance, mutual detoxication, mutual restraint, mutual inhibition, and antagonism between two drugs [1–3]. Amongst them, the mutual detoxication and mutual restraint have the same essence, and their purposes are both mainly used to reduce the toxicity of toxic TCM [1–3]. In recent years, under the guidance of TCM theory, the researches on the mechanisms of attenuated toxicity of toxic Chinese medicine such as Aconiti Radix, Aconm Lateralis Radix Praeparaia, arsenic trioxide etc. have become one of the hotspots of academic concern [4–8].

Radix *Tripterygium wilfordii* Hook F (LGT) is as a medicinal herb first seen in the ‘Shen Nong’s Materia Medica’ in Han dynasty of China with over 2000 years of medicinal history [9]. LGT has outstanding curative effects on diverse diseases including a variety of cancers [10–15], rheumatoid arthritis [16], systemic lupus erythematosus [17], glomerulonephritis [18], diabetic nephropathy [19], etc. However, LGT is also a potently toxic medicine, and often induces severe multiple organs toxicities, especially the liver toxicity [20–22], which has seriously limited its therapeutic efficacy and clinical application.

Herba *Lysimachia christinae* Hance (JQC) is traditionally seen as a dampness removing and jaundice-eliminating medicine, which was first recorded on ‘A Supplement to the Compendium of Materia Medica’ in the Qing dynasty of China, and has multiple biological activities including protection against acute alcohol-induced liver injury by involving the inhibition of oxidative stress damage [23], regressing pre-established cholesterol gallstone [24], anticholecystitis and cholagogic properties [25], hypuricemic effects [26], antioxidation [27], anti-inflammation [28] etc. In Chinese folk medicine, when taking LGT poisoning, JQC is often used to alleviate LGT-induced toxicity [9,29]. In addition, TCM theory holds that ‘JQC can prohibit LGT-induced poison’, that is to say, their combined administration belongs to the mutual detoxication of seven emotions in the compatibility [29]. However, to date, the compatibility detoxication mechanisms remain unknown.

Given that the liver is the major toxic target organ for LGT [9], our previous study [30] has conducted and demonstrated that compatibility with JQC administration inhibits the hepatotoxicity of LGT on physiological mice with a mechanism involved in enhancing antioxidant function. Considering that the drugs are mainly used on the patients rather than the normal ones in clinic and LGT has outstanding anticancers such as leukemia, breast cancer, lung cancer, prostate cancer, gastric cancer, cervical cancer [10–15], so we next aimed to observe the compatibility detoxication mechanisms of LGT by JQC on tumor pathological mice. Moreover, numerous studies [31–35] have reported that NF-E2-related factor 2 (Nrf2), as an important antioxidant transcription factor, plays a vital role in regulating the constitutive and inducible expression of many antioxidative and detoxifying enzymes to restore cellular redox homeostasis. Furthermore, increasing studies [33–35] have reported that many types of hepatotoxicity induced by hepatotoxicants such as carbon tetrachloride (CCl_4_), acetaminophen, or ethanol, are related to the imbalance of Nrf2, and enhancing Nrf2 activation is an important mechanism of drug’s prevention and treatment of hepatotoxicity. However, until now, whether the detoxication mechanisms of LGT via compatibility with JQC involve the activation of Nrf2 remains unknown.

Thus, the present study is undertaken to observe the detoxication mechanisms of LGT through compatibility with JQC in S180 tumor-bearing mice by involving Nrf2-mediated antioxidant signals. In addition, influence of compatibility on antitumor activity was also investigated here.

## Materials and methods

### Experimental animals

Considering that there is a gender difference in the toxicity of LGT, and that estrogens in female animals may probably interfere with the effects of drugs, male mice are used as research animals in the present study [36–38]. Kunming (KM) male mice (18–22 g) were obtained from Experimental Animal Center of Henan Province (Zhengzhou, China). Animals were given rodent laboratory chow and water *ad libitum* and maintained under controlled conditions with a temperature of 22 ± 1°C, relative humidity of 60 ± 10%, and a 12/12-h light/dark cycle (lights on at 7:00 a.m.). All the procedures were in strict accordance with the P.R. China legislation on the use and care of laboratory animals and guidelines formulated by the Institute for Experimental Animals of Henan University of Chinese Medicine. The procedures were approved by the university committee for animal experiments.

### Cell lines

Mouse S180 tumor cells were collected from S180 tumor-bearing mice which were purchased from the Institute of Chinese Materia Medica, China Academy of Chinese Medical Sciences (Beijing, China). Mouse S180 tumor cells were maintained in the peritoneal cavities of male KM mice in the Laboratory of Experimental Animals of Henan University of Chinese Medicine (Zhengzhou, China).

### Reagents

Kits including alanine/aspartate transaminase (ALT/AST), malondialdehyde (MDA), glutathione-s transferase (GST), glutathione peroxidase (GPx), superoxide dismutase (SOD), and catalase (CAT) were all purchased from Nanjing Jiancheng Bioengineering Institute (Nanjing, China). Mouse tumor necrosis factor-α (TNF-α) and interleukin (IL)-10 ELISA kits were provided by Boster Biological Technology (Wuhan, China).

### Preparations of extracts

The roots of LGT were provided by Taining County of Fujian province (Taining, China). JQC originates in Sichuan Province of China, and was purchased from a traditional drug store, Henan Materia Medica Chain Co., Ltd. (Zhengzhou, China). LGT and JQC were both identified by Prof Sui-Qing Chen (Pharmacognosy Department, Henan University of Chinese Medicine, Zhengzhou, China). Our previous research has confirmed that the combination with JQC administration in the mass ratio of 4/1~1/4 (LGT/JQC: 4/1, 2/1, 1/1, 1/2, and 1/4) attenuated LGT-induced subacute hepatotoxicity in the physiological state of mice, and the best ratio of such effect was 2/1 [39]. Therefore, this study selected the same ratios, 4/1~1/4 (LGT/JQC), to further investigate the mutual detoxication mechanisms of LGT and JQC under pathological conditions of the tumor. It has been reported that ethyl acetate has a good ability to dissolve triptolide (TP), the major active ingredient contained in LGT, and ethyl acetate is often used as an extraction solvent for LGT in many literatures [40–43]. Therefore, in the present study, we chose ethyl acetate as an extraction solvent to prepare all the extracts by conventional reflux extraction. The extraction yields of LGT and its compatibility with JQC (LGT/JQC: 4/1, 2/1, 1/1, 1/2, and 1/4) were 29.70, 27.25, 42.95, 39.28, 34.03, and 44.31 mg/g raw medicinal materials, respectively. Considering that TP has been confirmed as not only the major active chemical component of LGT but also the toxic/hepatotoxic one of it [44]. We measured the content of TP in the extracts before and after compatibility. The contents of TP in above extracts were 10.605, 2.013, 0.783, 2.031, 2.827, and 0.167 mg/g, respectively, as assayed by the HPLC analysis.

### Treatment protocol

According to the literature method with a slight modification, S180 ascites tumor cells were inoculated into the right armpit of mice to prepare S180 solid tumor model [45]. One day after inoculation, mice, except for the normal (non-tumor inoculated) animals, were randomly divided into seven groups with ten mice each. Mice in groups of the normal (without tumor inoculation or drug administration) and the control (only tumor-inoculated but without drug administration) received daily oral administration of 0.5% (5 g/l) sodium CM-cellulose (CMC-Na; 0.2 ml/10 g). Mice in the other six groups received extracts from LGT and its compatibility JQC (LGT/JQC: 4/1, 2/1, 1/1, 1/2, and 1/4) by intragastric administration (*ig*) for 12 days starting from 24 h after tumor inoculation. The doses of LGT and its compatability with JQC were all 60 mg/kg according to LGT at the same equivalent. Regardless of whether LGT was used alone or in combination, the dosage for animals refers to the LGT dose rather than the dose of the mixture. As a result, the mixed drug group and the LGT alone group were at the same level in terms of the LGT dose, and therefore they were comparable. After treatment, mice were killed by cervical dislocation after peripheral blood samples, livers, and tumors were collected at 24 h after the last administration. Serum samples were collected for the analysis of biochemical indicators including ALT and AST, and hepatic tissues were used for the analysis of the histological observation, lipid peroxide (LPO), glutathione (GSH), TNF-α, and IL-10 levels, determination of GSH-related and antioxidant enzymes, Nrf2 protein expression and Nrf2-regulated downstream genes such as heme oxygenase-1 (HO-1), NAD(P)H:quinone oxidoreductase-1 (NQO1), and glutamate cysteine ligase catalytic subunit (GCLC). The tumors were weighed, arrayed in line on paper, and pictures were taken. The tumor inhibition ratio (IR) was calculated by the formula of IR = [(C − T)/C] × 100%, where C and T are the mean tumor weights of the control group and the treated group, respectively.

### Assay for serum ALT and AST

Blood samples were obtained from mice of all groups (ten mice per group) for the analysis of serum biochemical indicators including ALT and AST by the commercial kits (Nanjing Jiancheng Bioengineering Institute, Nanjing, China) in accordance with the manufacturer’s protocols.

### Histological observation

After fixation in 10% formalin, the livers were examined for size, color changes, and hemorrhage. Slices of livers were cut into small pieces and histological sections were stained with Hematoxylin and Eosin (H&E) for observation under 200 times with light microscope.

### Assay for hepatic LPO levels

Hepatic tissues were homogenized in cold physiological saline, respectively. LPO was determined by the previous reported method [46]. MDA is the end product of LPO and serves as a means of quantitating LPO. MDA reacts with 2-thiobarbituric acid (TBA) to generate a pink-colored product, which has an absorbance at 532 nm. LPO level was expressed as micromoles of MDA per milligram of protein based on tissue protein concentration.

### Assay for hepatic GSH levels, GST, GPx, SOD, and CAT levels

Hepatic tissues’ GSH levels, GST, GPx, SOD, and CAT activities were analyzed by the commercial kits (Nanjing Jiancheng Bioengineering Institute, Nanjing, China) in accordance with the manufacturer’s protocols, and the results were all calculated based on tissue protein concentrations.

### Assay for hepatic IL-10 and TNF-α levels

Hepatic IL-10 and TNF-α levels were analyzed by the commercial kits (Boster Biological Technology, Wuhan, China) in accordance with the manufacturer’s protocols, and the results were all calculated based on tissue protein concentrations.

### Western blot analysis

Hepatic proteins were separated as described in the Nuclear Extraction Reagent Kit (Pierce, U.S.A.). The protein concentrations were measured, and all the samples in the same experiment were normalized to equal protein concentration. Protein samples were isolated by SDS/PAGE gel electrophoresis and transferred on to a PVDF membrane, and then incubated with the appropriate combination of primary and secondary antibodies, followed by ECL detection and quantitation using an image analysis program. The gray densities of the protein bands were normalized by using Lamin B density as internal control, and the results were further normalized to control.

### Real-time PCR analysis

Total RNA was extracted from hepatic tissue using TRIzol reagent following the manufacturer’s instructions. cDNA was synthesized and real-time PCR was conducted as described in kits. The PCR primer sequences were as follows: HO-1 forward 5′-ACGCATATACCCGCTACCTG-3′, reverse 5′-CCAGAGTGTTCATTCGAGCA-3′ (174 bp product); NQO1 forward 5′-GCGTCTGGAGACTGTCTGGG-3′, reverse 5′-CGGCTGGAATGGACTTGC-3′ (170 bp product); GCLC forward 5′-AACACAGACCCAACCCAGAG-3′, reverse 5′-CCGCATCTTCTGGAAATGTT-3′ (201 bp product); and glyceraldehyde-3-phosphate dehydrogenase (GAPDH) forward 5′-CAAGGTCATCCATGACAACTTTG-3′, reverse 5′-GGGCCATCCACAGTCTTCTG-3′ (90 bp product). Relative expression of target genes was normalized to GAPDH, analyzed by 2^−ΔΔ*C*^_t_ method and given as ratio compared with the control.

### Statistical analysis

The results were presented as mean ± S.D. The differences amongst experimental groups were compared by one-way ANOVA followed by Least Significant Difference (LSD) (*P*<0.05) using the SPSS (Statistics Package for Social Science) program Version 17.0.

## Results

### Compatibility with JQC exerted detoxication effects on LGT-induced hepatotoxicity, and the converted dose of TP in each extract

**Figure 1:**
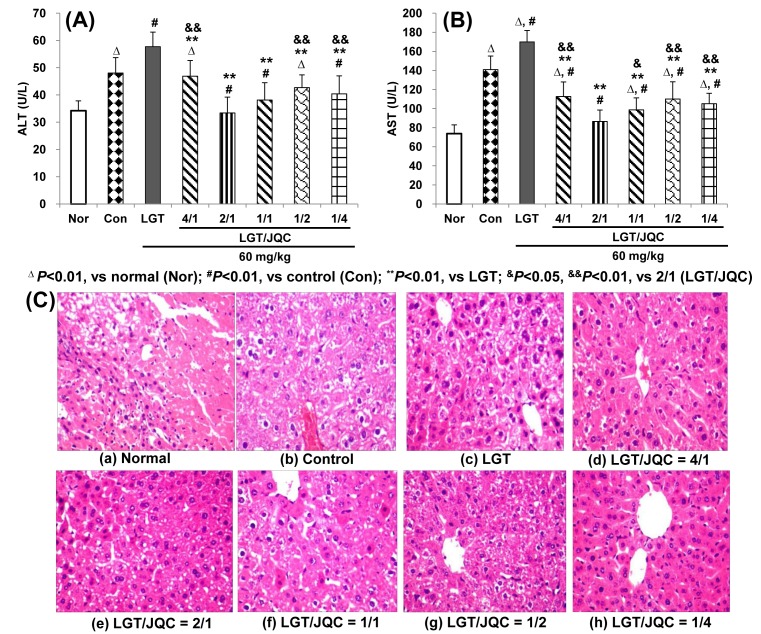
Compatibility with JQC exerted detoxication effects on LGT-induced hepatotoxicity. Effects of compatibility with JQC on serum ALT (**A**) and AST (**B**) levels, and hepatic histopathology (**C**) in LGT-exposed S180-bearing mice. Data are presented as mean ± S.D. (*n*=10). ^Δ^*P*<0.01 compared with normal (Nor); ^#^*P*<0.01 compared with control (Con); **P<0.01 compared with LGT; ^&^*P*<0.05, ^&&^*P*<0.01 compared with 2/1 (LGT/JQC).

Serum ALT and AST levels are sensitive parameters of liver function [35]. In the current study, compared with the normal group, S180 tumor-bearing induced the significant elevation of serum ALT and AST levels (both *P*<0.01) (Figure 1A,B). Compared with the control group, LGT administration further elevated the S180 tumor-increased ALT and AST levels (both *P*<0.01) (Figure 1A,B), indicating LGT evoked the hepatotoxicity under S180 tumor pathological state of mice. Compared with the LGT alone administration group, compatibility with JQC at the ratios (LGT/JQC from 4/1 to 1/4) all significantly reduced LGT-elevated serum ALT and AST levels (all *P*<0.01) (Figure 1A,B), which demonstrated that compatibility with JQC at all the ratios exerted detoxication effect on LGT-induced hepatotoxicity from serum biochemical analysis. In addition, the compatibility of LGT with JQC had significantly lower levels of serum ALT and AST in the ratio 2/1 group than those in the 4/1, 1/2, and 1/4 groups (all *P*<0.01), and significantly lower serum AST level than that in the 1/1 group (*P*<0.05), which indicated that the 2/1 could be the optimal ratio of detoxication effect. In addition, of the above five ratios, only when LGT/JQC was administered at a ratio of 2/1, both ALT and AST of tumor-bearing mice returned to normal animal levels.

Moreover, hepatic histological analysis demonstrated LGT alone administration hepatocytes hydropic degeneration, while compatibility with JQC at all above proportions, the abnormal changes conspicuously decreased or even disappeared (Figure 1C), which further corroborated that compatibility with JQC exerted detoxication effect on LGT-induced hepatotoxicity from pathology analysis.

In addition, depending on the amount of TP in the extract, the dose of extract is converted into the dose of TP, and then we can get the converted TP dose. Thus, the converted TP doses of the compatibility extracts (LGT alone and LGT compared with JQC in the ratios of 4/1, 2/1, 1/1, 1/2, and 1/4) are 636.3, 120.8, 47.0, 121.9, 169.6, 10.0 μg/kg, respectively.

### Compatibility with JQC up-regulated Nrf2 and it-target downstream genes expression

**Figure 2:**
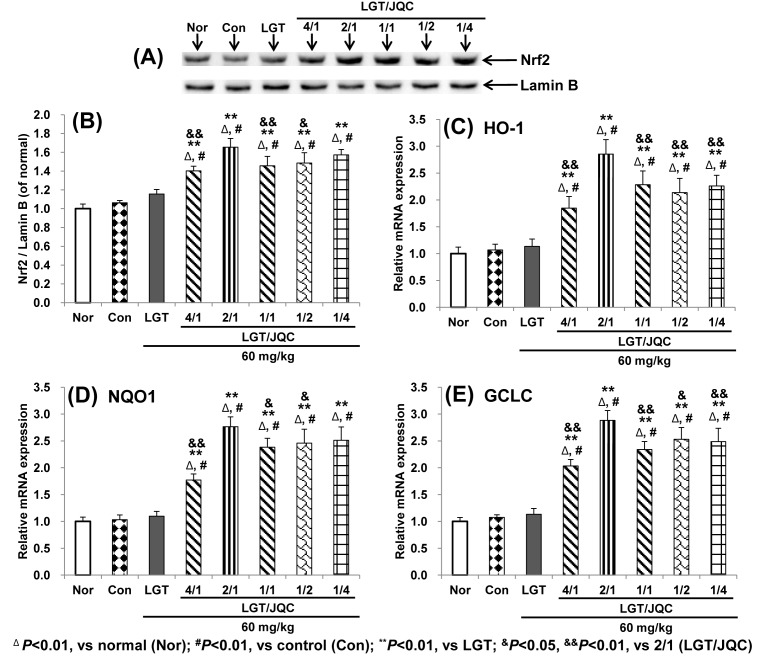
Compatibility with JQC up-regulated Nrf2 and it-target downstream genes expression. Effects of compatibility with JQC on hepatic protein expression of Nrf2 (**A**,**B**) and mRNA expression of it-regulated genes including *HO-1* (**C**), *NQO1* (**D**), and *GCLC* (**E**) in LGT-exposed S180-bearing mice. Data are presented as mean ± S.D. (*n*=10). ^Δ^*P*<0.01 compared with normal (Nor); ^#^*P*<0.01 compared with control (Con); **P<0.01 compared with LGT; ^&^*P*<0.05, ^&&^*P*<0.01 compared with 2/1 (LGT/JQC).

It is reported that Nrf2 nuclear translocation is indispensable for activating various antioxidative genes such as *HO-1, NQO1*, and *GCLC* as key antioxidant transcription factors [32–35,40]. Western blot analysis demonstrated that both S180 tumor-bearing and LGT alone administration slightly enhanced the protein expression of hepatic nuclear Nrf2, but there was no significant difference, while compatibility with JQC in the ratios (LGT/JQC from 4/1 to 1/4) all remarkably up-regulated the protein expression of hepatic nuclear Nrf2 compared with LGT alone administration group (all *P*<0.01) (Figure 2A,B), suggesting that compatibility with JQC administration promoted nuclear translocation of Nrf2. In addition, the compatibility of LGT with JQC had significantly higher level of Nrf2 protein expression in the ratio 2/1 group than those in the 4/1, 1/1, and 1/2 groups (*P*<0.01, *P*<0.01, and *P*<0.05, respectively), and slightly higher than that in the 1/4 group but no significant difference.

Further analysis by real-time PCR demonstrated that both S180 tumor and LGT administration also slightly promoted the mRNA expression of Nrf2-target downstream antioxidative genes such as *HO-1, NQO1*, and *GCLC* but there was no significant difference, while compatibility with JQC in the ratios (LGT/JQC from 4/1 to 1/4) all remarkably up-regulated the mRNA expression of these target genes compared with LGT alone administration group (all *P*<0.01) (Figure 2C–E). These results indicated that the promotion of Nrf2 nuclear translocation and activation of Nrf2 downstream target antioxidative genes could be involved in the attenuated effect of compatibility with JQC in each ratio from 4/1 and 1/4 on liver toxicity induced by LGT under S180 tumor pathological state of mice. In addition, the compatibility of LGT with JQC had significantly higher mRNA expression levels of Nrf2 target genes *HO-1* and *GCL*C in the ratio 2/1 group than those in all the other ratio groups (for HO all *P*<0.01, and for GCLC *P*<0.01, *P*<0.01, *P*<0.05, and *P*<0.01, respectively), and higher NQO1 in the ratio 2/1 group than those in the 4/1, 1/1, and 1/2 groups (*P*<0.01, *P*<0.05, and *P*<0.05, respectively) and slightly higher than that in the 1/4 group but no significant difference.

### Compatibility with JQC reversed LGT-induced abnormal levels of hepatic LPO and antioxidant indicators

**Figure 3:**
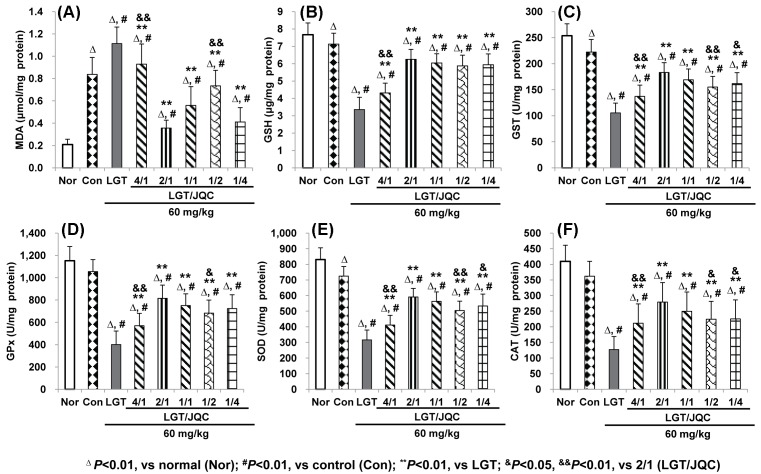
Compatibility with JQC reversed LGT-induced abnormal levels of hepatic LPO and antioxidant indicators. Effects of compatibility with JQC on hepatic MDA (**A**) and antioxidant indicators including GSH (**B**), GST (**C**), GPx (**D**), SOD (**E**), and CAT (**F**) in LGT-exposed S180-bearing mice. Data are presented as mean ± S.D. (*n*=10). ^Δ^*P*<0.01 compared with normal (Nor); ^#^*P*<0.01 compared with control (Con); **P<0.01, compared with LGT; ^&^*P*<0.05, ^&&^*P*<0.01 compared with 2/1 (LGT/JQC).

It has been confirmed that MDA is a sensitive indicator of LPO damage [47], and GSH, GST, GPx, SOD, and CAT are important endogenous antioxidants or antioxidative enzymes in the body [45,47–50]. In the present study, compared with the normal group, S180 tumor-bearing caused a significant increase in liver MDA (*P*<0.01) (Figure 3A), and significant reduction in GSH, GST, and SOD (all *P*<0.01) (Figure 3B,C,E), but no significant difference in GPx and CAT (Figure 3D,F). Compared with the control group, LGT administration significantly elevated the MDA level (*P*<0.01) (Figure 3A), and reduced GSH, GST, GPx, SOD, and CAT levels (all *P*<0.01) (Figure 3B–F) in hepatic of S180 tumor-bearing mice. Compared with the LGT alone administration group, compatibility with JQC in the ratios (LGT/JQC from 4/1 to 1/4) all significantly reduced LGT-elevated MDA level (*P*<0.01) (Figure 3A), and elevated LGT-decreased GSH, GST, GPx, SOD, and CAT levels (all *P*<0.01) (Figure 3B–F) in hepatic of S180 tumor-bearing mice. These results demonstrated that the inhibition of LPO and enhancement of endogenous antioxidants or antioxidative enzymes could be involved in the attenuated effect of compatibility with JQC in each ratio from 4/1 and 1/4 on liver toxicity induced by LGT under S180 tumor pathological state of mice.

### Compatibility with JQC reversed LGT-induced abnormal levels of partial inflammation related indicators

**Figure 4:**
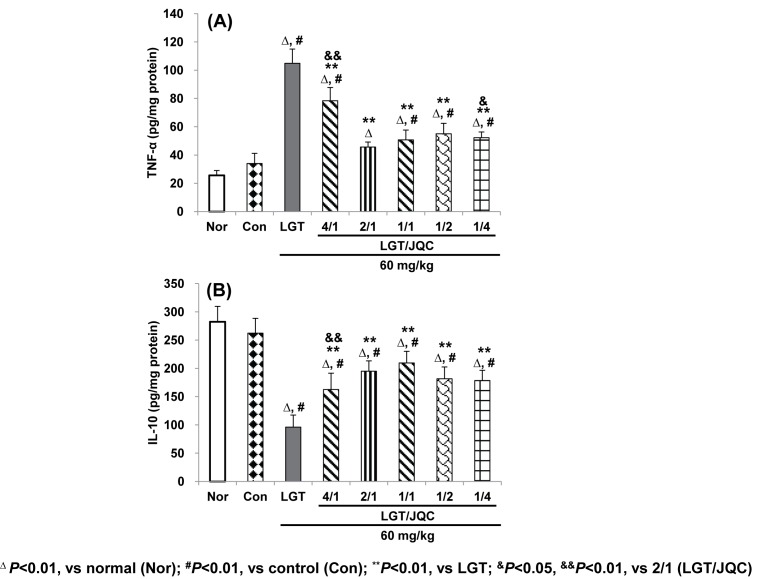
Compatibility with JQC reversed LGT-induced abnormal levels of partial inflammation related indicators. Effects of compatibility with JQC on hepatic partial inflammation-related indicators including TNF-α (**A**) and IL-10 (**B**) in LGT-exposed S180-bearing mice. Data are presented as mean ± S.D. (*n*=10). ^Δ^*P*<0.01 compared with normal (Nor); ^#^*P*<0.01 compared with control (Con); **P<0.01 compared with LGT; ^&^*P*<0.05, ^&&^*P*<0.01 compared with 2/1 (LGT/JQC).

TNF-α and IL-10, as intracellular key inflammatory mediators, participate during many types of hepatotoxicity [51–55]. The results of this study showed that S180 tumor-bearing slightly increased hepatic TNF-α and decreased IL-10 levels (Figure 4A,B), but no statistical difference. LGT administration significantly increased hepatic TNF-α and decreased IL-10 levels (both *P*<0.01) (Figure 4A,B) compared with the control group, while compatibility with JQC in the ratios (LGT/JQC from 4/1 to 1/4) all significantly reduced LGT-increased TNF-α and LGT-decreased IL-10 levels (all *P*<0.01) (Figure 4A,B) in hepatic of S180 tumor-bearing mice. These results demonstrated that the anti-inflammatory reactions (at least from TNF-α and IL-10) could be partially involved in the attenuated effect of compatibility with JQC in each ratio from 4/1 and 1/4 on liver toxicity induced by LGT under S180 tumor pathological state of mice.

### Compatibility with JQC augmented LGT’s antitumor activity

**Figure 5:**
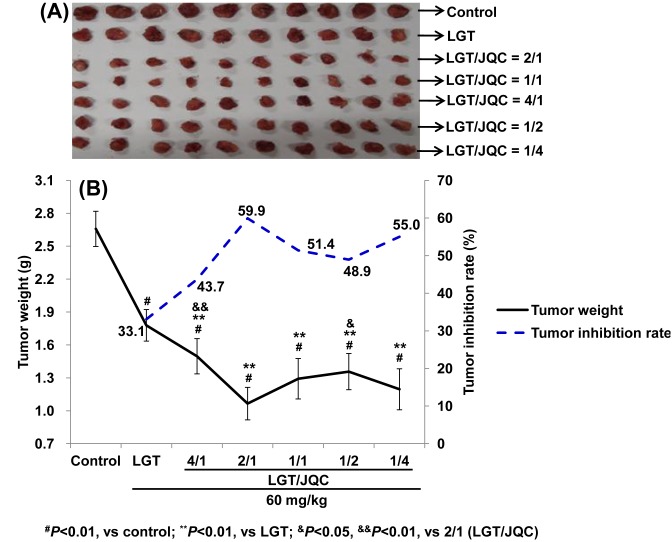
Compatibility with JQC augmented LGT’s antitumor activity. Effects of compatibility with JQC on the antitumor activity ((**A**) tumor picture and (**B**) tumor weight and inhibition rate) in LGT-exposed S180-bearing mice. Data are presented as mean ± S.D. (*n*=10). ^#^*P*<0.01 compared with control; **P<0.01 compared with LGT; ^&^*P*<0.05, ^&&^*P*<0.01 compared with 2/1 (LGT/JQC).

The effect of compatibility with JQC administration on the antitumor activity of LGT in S180 tumor-bearing mice was shown in Figure 5A,B. The results of the present study demonstrated that LGT alone administration significantly decreased tumor weight of S180-bearing mice (*P*<0.01) with the tumor inhibition rate 33.1%, while LGT-decreased tumor weight was further decreased by compatibility with JQC at the ratios of 4/1, 2/1, 1/1, 1/2, and 1/4 with the elevated inhibition rates of 43.7, 59.9, 51.4, 48.9, and 55.0%, respectively (all *P*<0.01) (Figure 5A,B).

## Discussion

Serum ALT and AST are sensitive indicators of liver function and their obvious elevation commonly indicates liver toxicity [35]. In the present study, compared with the normal group, serum ALT and AST levels were significantly elevated in the control group of mice, indicating tumor-bearing induced liver damage, which was consistent with the results reported in many literatures [45,47–50]. After LGT administration to the tumor-bearing mice, those above elevation were further significantly elevated, which indicated that LGT aggravated the hepatotoxicity in tumor-bearing mice. Compatibility with JQC in the five ratios administration all significantly reversed LGT-elevated serum ALT and AST levels of tumor-bearing mice, indicating that compatibility with JQC in all the given ratios exerted detoxication on LGT-induced hepatotoxicity under tumor-bearing pathological state of mice. Further liver pathological analysis also reflected the detoxication of LGT in the pathological state of the tumor with the compatibility of JQC. However, the effect of JQC alone on tumor-bearing mice was not tested, which is a limitation of the present study.

It is worth mentioning that the ratio of 2/1 in the present study is the best ratio of the detoxification effect of LGT and JQC, with evidence of lower ALT and AST levels in the tumor state compared with the others for ratios. But why is this? One of the main possible reasons is probably that compatibility affects the content of TP, a major toxic and active chemical component of LGT. From the analysis of converted doses, we hypothesized that the dose reduction in the toxic ingredient TP may be one of the main reasons for the compatibility attenuation and the low TP dose at the 2/1 ratio, which may be an important reason for its better effect. In addition, in fact, a study [39] we recently reported has initially confirmed that the chemical component that contributes most to the detoxication of LGT by the combination of JQC is TP, which is consistent with the results of the TP conversion dose estimation in the present study. As for how compatibility with JQC affects other chemicals in the LGT and thus affects such attenuated effects, further study is needed.

Considering that the hepatotoxicity induced by LGT was reported to involve LPO and oxidative stress damage [20,51,52] and JQC was reported to exert antioxidant capacity [28], we next observed whether the inhibition of LPO, the endogenous antioxidants such as GSH, GST, GPx, SOD, and CAT, antioxidative key transcription factor Nrf2 and its downstream antioxidative target genes are involved in the attenuation of LGT compatibility with JQC. Our results demonstrated that tumor bearing led to the significant elevation of MDA and reduction in GSH, GST, and SOD levels, but no significant difference in GPx and CAT levels, suggesting tumor bearing induced LPO damage and lower levels of antioxidants (at least reflected in GSH, GST, and SOD), which was almost the same as reported in the literatures [45,47–50]. After LGT administration to the tumor-bearing mice, the significantly elevated MDA and reduced GSH, GST, GPx, SOD, and CAT levels were seen compared with the tumor control group, indicating that LGT induced LPO damage and lower levels of antioxidants of tumor-bearing mice. Fortunately, compatibility with JQC at the five ratios all significantly reversed LGT-induced LPO damage and lower levels of antioxidants in tumor-bearing mice. Furthermore, compatibility with JQC at the five ratios all significantly promoted nuclear translocation of Nrf2 and activated *HO-1, NQO1*, and *GCLC* mRNA expression, suggesting that the activation of Nrf2 and it-target downstream genes could involve the protection of compatibility with JQC against LGT-induced hepatotoxicity in tumor-bearing mice. In fact, it has been reported that chlorogenic acid, quercetin, and rutin, which are all the active chemical ingredients of JQC, all reported to protect hepatotoxicity induced by acetaminophen, carbon tetrachloride (CCl_4_), ethanol, hydrogen peroxide, or cholestatic by activating Nrf2 and its downstream target genes [31–35]. Therefore, we speculate that the attenuated effect of compatibility with JQC on LGT-induced hepatotoxicity may be related to the activation of Nrf2 and its downstream target genes by active ingredients such as chlorogenic acid, quercetin, and rutin contained in JQC. However, in-depth mechanism needs further study. Additionally, in fact, to a certain extent, promoting Nrf2 nuclear aggregation will in turn promote up-regulation of mRNA expression by the downstream genes it targets, such as *HO-1, NQO1*, and *GCLC*. Therefore, in the present study, we measured Nrf2 protein expression and mRNA expression of Nrf2-targetted downstream genes such as *HO-1, NQO1*, and *GCLC*, but neither *Nrf2* mRNA expression nor protein expression of these downstream genes targetted by Nrf2 was detected. Of course, if we also examine the mRNA expression of Nrf2 and the protein expression of downstream genes targetted by Nrf2, such as *HO-1, NQO1*, and *GCLC*, to a certain extent, explain the role of Nrf2 in the mechanism of compatibility detoxication of the present study, it may be more comprehensive. Therefore, the expression of *Nrf2* mRNA and proteins expressed by downstream genes targetted by Nrf2 such as *HO-1, NQO1*, and *GCLC* have not been determined, which is one of the limitations of the present study.

Interestingly, some latest studies [53–57] have shown that activation of Nrf2 not only enhanced antioxidant activity, but also suppressed inflammatory responses triggered by some diseases including hepatotoxicity. Furthermore, in recent years, a variety of studies [58–62] have also shown that hepatotoxicity caused by multiple hepatotoxicants including acetaminophen, ethanol, CCl_4_, d-galactosamine/lipopolysaccharide, thioacetamide etc. was involved in abnormal inflammatory reaction, often evidenced by the excessive increase in pro-inflammatory cytokine TNF-α and excessive decrease in anti-inflammatory cytokine IL-10. Moreover, several studies have reported that the hepatotoxicity caused by LGT was related to its inflammatory response [20,52,63]. Therefore, we next investigated the effects of compatibility with JQC on LGT-induced hepatotoxicity by analyzing inflammatory mediators including TNF-α and IL-10. Our results showed that LGT administration induced abnormal inflammatory reaction, at least evidenced by significantly elevated hepatic TNF-α and reduced IL-10 levels (both *P*<0.01) (Figure 4A,B) compared with the control group. Fortunately, after compatibility with JQC treatment in the given five ratios, the abnormalities of these two inflammatory mediators were significantly reversed, suggesting the anti-inflammatory reaction by at least TNF-α and IL-10 could partially involve the protection of compatibility with JQC against LGT-induced hepatotoxicity in tumor-bearing mice. In fact, it has also been reported that the inflammatory response associated with LGT-induced hepatotoxicity also involved excessive levels of IL-2 and prostaglandin (PG) E_2_ [63]. However, this study did not address the inflammatory mediators of IL-2 and PGE_2_, which may be a limitation of this study.

After elucidating the detoxication mechanism of LGT via compatibility with JQC, we further investigated the influence of compatibility on the antitumor activity of LGT. Our results showed that the compatibility of JQC significantly increased the antitumor effect of LGT in tumor-bearing mice, and the ratio 2/1 was also the best for such synergism. We speculate that this effect may also be associated with the inhibition of LPO and inflammation as well as enhanced antioxidant activity based on Nrf2 signaling.

Recently, we have just reported that the median lethal dose (LD_50_) of acute toxicity to mice was 517 mg/kg when LGT was used alone, and the LD_50_ of LGT increased to 889 mg/kg after the optimal ratio of 2/1 to JQC, indicating that the acute toxicity was decreased by 72% [39]. Even so, in view of such LD_50_ values, it shows that there are still security risks of medication.

Collectively, through compatibility with JQC exerted detoxication effect on LGT-induced hepatotoxicity and the mechanisms could be at least partly attributed to up-regulation of Nrf2 and its downstream signals, thereby enhancing antioxidant defenses, and inhibiting lipid peroxidation, oxidative stress and inflammation. Additionally, at the ratio 2/1 (LGT/JQC) exerted the strongest effects on both detoxication and synergism. Based on the results we have obtained, we can provide some tips or suggestions for clinical use of LGT. First of all, when we use LGT clinically, we can consider the combination of JQC with 2/1 ratio, which will reduce the toxicity and enhance the antitumor activity. In fact, it has been reported that clinically, LQT poisoning can be detoxified with JQC [29], which to a certain extent also supports the clinical implications of our results. In addition, we can also consider the use of combination with JQC to reduce the intake of LGT, thereby reducing the risk of poisoning, but its efficacy is not reduced, although this consideration requires more evidence to support.

## Abbreviations

ALT: alanine transaminase
AST: aspartate transaminase
CAT: catalase
GAPDH: glyceraldehyde-3-phosphate dehydrogenase
GCLC: glutamate cysteine ligase catalytic subunit
GPx: glutathione peroxidase
GST: glutathione-s transferase
HO-1: heme oxygenase-1
IL: interleukin
IR: inhibition ratio
JQC: Herba *Lysimachia christinae* Hance
KM: Kunming
LGT: Radix *Tripterygium wilfordii* Hook F
LPO: lipid peroxide
MDA: malondialdehyde
NF-E2: nuclear factor erythroid 2
NQO1: NAD(P)H: quinone oxidoreductase-1
Nrf2: NF-E2-related factor 2
PG: prostaglandin
SOD: superoxide dismutase
TCM: traditional Chinese medicine
TNF-α: tumor necrosis factor-α
TP: triptolide
